# Poor-Law Institutions and Nurse-Training
*The first section of this article appeared in The Hospital on November 4, 1916, p. 103.


**Published:** 1916-11-11

**Authors:** 


### THE MATRONS' AND SISTERS' DEPARTMENT.

### II. POOR-LAW INSTITUTIONS AND NURSE-TRAINING.*

The clinical practice at these institutions is nowadays excellent and varied. It is true that a large proportion of the cases treated are of less acute type, but amongst them are found many forms of disease seldom treated in general hospitals, where the admissions per bed are at a higher rate or where (in other words) the average duration of patients' stay in the wards is shorter. The educational value, to the nurse, of her patients is certainly as great. The surgical operations are proportionately fewer and are simpler in character; this, however, is by no means necessarily to the disadvantage of nurse-teaching because, in the operating theatre as in the wards, the nurse takes a more prominent and active share in what is going on. In the absence of resident medical officers, students, and instrument-men who tend, in a busy routine, to keep limited the duties of the nurse and to relegate her to the background, she takes a relatively more important position and *does* more. Being given greater responsibility with regard to such matters as sterilisation, dressings, saline infusions, and hypodermic injections, she gets a wider practice and an observance and readiness in emergencies which are invaluable to her in after-life.

The nurse, like the medical student, cannot in the course of her training see and learn everything; specialisation is, in either case, to be discouraged. To the nurse, therefore, the absence from her training-school of highly specialised *x*-ray, electrical, and pathological departments, etc., is unimportant; nor has an overworked out-patient department (with the exception of surgical casualty work) any great instructional value to her. The infirmary nurse may, however, gain some useful experience in the wards for female mental cases, the lying-in wards, and the wards for the tuberculous and for children and infants. Often she stays on after gaining her training certificate and prepares for such examinations as those of the Incorporated Society of Trained Masseuses or the Central Midwives Board, for which instructional facilities are given her; teaching in dispensing is sometimes obtainable.

It is obvious that the infirmary nurse is brought into a somewhat closer and more undisturbed relationship with her patients. These are not now exclusively, or even principally, of what has been termed the "pauper class"; they are not even largely chronic or senile cases drafted from the workhouse. The "daddy" and "granny" often erroneously still associated with Poor-Law nursing are now commonly relegated to, and very well tended in, workhouse sick-wards, and not in those of the nurse-training school. In these latter the association between nurse and patient entails nothing exceptional or detrimental to the nurse; in fact she may have opportunities of observing phases of her patients' mentality which are far from being devoid of educational value, and should assist her in forming her character and in giving her a sympathetic and balanced judgment. The infirmary-trained nurse often proves singularly tactful, agreeable, and reasonable, qualities which are invaluable in private nursing, and go far towards preventing the friction and restraint which so readily arise in a household suddenly disturbed by illness.

The domestic and social relationships of the infirmary nursing schools have steadily improved and continue to improve. Their tone, depending as it must upon the wise control of a good matron, and the effects of their disciplined, semi-collegiate life, are excellent. The dieting and the care of the health of the nurses have reached a high standard, whilst Guardian Boards have come generally to recognise not ungenerously the need for holidays, for exercise, and for means of (at any rate) simple forms of recreation and amusement.

### ROUND THE HOSPITALS.

* The first section of this article appeared in The Hospital on November 4, 1916, p. 103.

